# The Immunomodulatory Potential of Concurrent High-Dose Radiotherapy and Immune Checkpoint Inhibitor Cemiplimab in Advanced Cutaneous Squamous Cell Carcinoma: Initial Results

**DOI:** 10.3390/jpm14060581

**Published:** 2024-05-29

**Authors:** Maria Chiara Lo Greco, Giorgia Marano, Roberto Milazzotto, Rocco Luca Emanuele Liardo, Irene Finocchiaro, Madalina La Rocca, Antonio Basile, Pietro Valerio Foti, Stefano Palmucci, Emanuele David, Stefano Pergolizzi, Corrado Spatola

**Affiliations:** 1 Radiation Oncology Unit, Department of Biomedical, Dental and Morphological and Functional Imaging Sciences, University of Messina, 98122 Messina, Italy; giorgiamarano@gmail.com (G.M.); irene.finocchiaro.com@gmail.com (I.F.); madalina.larocca@gmail.com (M.L.R.); stefano.pergolizzi@unime.it (S.P.); 2 Radiation Oncology Unit, University Hospital Policlinico “G. Rodolico-San Marco”, 95123 Catania, Italy; r.milazzotto@policlinico.unict.it (R.M.); lucaliardo@hotmail.com (R.L.E.L.); cor_spatola@hotmail.com (C.S.); 3 Radiology I Unit, University Hospital Policlinico “G. Rodolico-San Marco”, 95123 Catania, Italy; basile.antonello73@gmail.com (A.B.); pietrofoti@hotmail.com (P.V.F.); spalmucci@unict.it (S.P.); david.emanuele@yahoo.it (E.D.); 4 Department of Medical Surgical Sciences and Advanced Technologies “G.F. Ingrassia”, University of Catania, 95123 Catania, Italy

**Keywords:** squamous cell cancer, high-dose radiotherapy, abscopal effect, cemiplimab

## Abstract

In this retrospective case series, we investigate the synergistic effect and the immunomodulatory potential of combination radiotherapy and immunotherapy on 11 patients affected by locally advanced or metastatic cutaneous squamous cell carcinoma (CSCC), treated at our institution between 2020 and 2023. The primary endpoints of this study are objective tumor response, assessed by Immunotherapy Response Evaluation Criteria in Solid Tumors (iRECIST), and time to treatment failure (disease progression). In all patients, surgery was deemed not amenable, due to its potential functional and aesthetic impact. Therefore, upon multidisciplinary agreement, radiotherapy and immunotherapy with cemiplimab were alternatively administered. After 6 months, an early objective tumor response was observed in 9/11 patients, with 17/20 cutaneous lesions (85%) presenting either a complete or partial response. Only 2/11 patients, with a total of 3/20 cutaneous lesions (15%), had stable disease. These benefits persisted at a longer follow-up (21.4 ± 9.7 months), with no patients presenting disease progression. Despite the retrospective nature of this study and small sample size, our experience highlights the ability of concomitant radiotherapy and cemiplimab to promote an early objective response in patients with advanced CSCC. Moreover, in our population, the clinical benefits were also related to a longer progression-free survival, without any safety alert reported.

## 1. Introduction

Cutaneous squamous cell carcinoma (CSCC) is the second most prevalent form of non-melanoma skin cancer (NMSC), accounting for 25% of cases and nearly 75% of NMSC-related deaths [1].

CSCC typically originates from epidermal keratinocytes and manifests as a spectrum of progressively advanced malignancies, ranging from precursor actinic keratosis to in situ carcinoma, followed by invasive and finally metastatic CSCC [2]. Because of this spectrum of conditions, the therapeutic approaches available for this malignancy also vary according to the severity of the stage.

The first-line treatment option for most CSCCs is surgery with adequate margins. However, in cases of inoperable disease or high post-operative risk, radiation therapy can be performed, offering satisfactory cosmetic and functional outcomes [3].

Radiation treatment can be performed with beams of electrons, photons, surface brachytherapy, or protons, as experimented in our center, for squamous cell carcinomas of the conjunctiva or porocarcinomas [4,5,6].

In locally advanced or metastatic CSCC, several systemic agents have been shown to have clinical benefits, including chemotherapy, epidermal growth factor receptor (EGFR)-targeted therapy, and immunotherapy with programmed cell death-1 (PD-1) inhibitors [7]. Nevertheless, the optimal approach in this setting remains unclear, with no established consensus on first-line systemic treatment.

Given the promising results of combining radiotherapy and immunotherapy for several malignancies such as advanced melanoma, head and neck district CSCC, and non-small-cell lung cancer [8,9,10,11], we performed a retrospective case series study, investigating the synergistic effect and immunomodulatory potential of this combination on 11 patients with locally advanced CSCC, treated at our institution between 2020 and 2023. The primary endpoints of this study are objective tumor response, assessed by Immunotherapy Response Evaluation Criteria in Solid Tumors (iRECIST), and time to treatment failure (disease progression). The feasibility of the combined treatment was evaluated using the Common Terminology Criteria for Adverse Events (CTCAE v4.0).

## 2. Materials and Methods

For this retrospective case series, 11 patients with locally advanced or metastatic CSCC treated at the University of Catania between 2020 and 2023 have been selected. The inclusion criteria were age > 18 years, single or multiple cutaneous lesions with a histological diagnosis of CSCC, clinical and radiological diagnosis of locally advanced or metastatic disease, radiotherapy treatment, and immunotherapy with cemiplimab. The patients’ characteristics are summarized in Table 1.

At baseline, all patients presented one or more ulcerated, patchy, papulonodular, papillomatous, or exophytic lesions, specifically in sun-exposed skin areas (the face, the scalp, and the backs of the hands) and other non-exposed areas (i.e., trunk, feet).

Biopsy and histological examination were performed to obtain diagnosis confirmation of CSCC. In detail, a total of 20 skin lesions were identified in a population of 11 patients.

Following this, additional imaging techniques such as contrast-enhanced Computed Tomography (CT) or Magnetic Resonance Imaging (MRI) were performed to assess the depth of invasion. Assessment of in-transit regional lymph node metastases was made with an accurate physical examination, ultrasonography, and PET-CT when needed. Tumor staging was established according to the TNM (tumor, node, and metastasis) American Joint Committee on Cancer (AJCC) criteria, with all patients being affected by either locally advanced or metastatic SCC.

Surgery was deemed not amenable due to its potential functional and aesthetic impact; therefore, radiotherapy and immunotherapy with cemiplimab were offered to the patients, upon multidisciplinary agreement.

To design a unique and tailored radiotherapy treatment, each patient underwent a simulation process through a CT scan (3–5 mm slice spacing) without contrast enhancement. Since the patient’s position must be the same for both initial simulation and subsequent treatment, different immobilization devices (based on tumor locations) were used to obtain an optimal setup.

After CT image acquisition, the treatment planning software Raystation^®^ (Ray Search Laboratories) version 12, was used for target volume definition and dose solutions. During the contour delineation phase, the gross tumor volume (GTV) was outlined, including the primary tumor and positive nodes (when present). The clinical target volume (CTV) was then delineated and successively expanded to obtain a planning target volume (PTV). After the delineation phase, a personalized plan was built up by the medical physicist, in synergy with the radiation oncologist, respecting the dose limits of any organ at risk (OAR) to minimize the side effects of the treatment. All patients received a 0.5 cm thick water bolus that had increased PTV coverage.

External-beam radiation therapy was delivered through an Elekta VERSA HD^®^ linear accelerator, using electron beams or photon beams through either a 3D-conformal technique (3D-CRT) or a static step-and-shot intensity-modulated technique (IMRT), based on the conformation of the target. During the entire course of radiotherapy treatment, patients were set up daily by using one sagittal and two lateral tattoos and lasers. A kilovoltage cone-beam computer tomography image-guided radiotherapy (KV CBCT IGRT) system was used two times a week to check patients’ setups. All treatments were performed on a HexaPod^®^ (ELEKTA) carbon fiber robotic treatment table capable of performing corrections in all 6 degrees of freedom. Patients with face or scalp lesions were monitored through daily optical surface-guided imaging (VisionRT–AlignRT^®^).

Different fractionation schedules were used, including conventionally fractionated radiation therapy (CFRT) or hypofractionated radiation therapy (HFRT). BED was calculated using an alpha/beta ratio of 10. Radiotherapy schedules are summarized in Table 2.

Cemiplimab 350 mg was administered intravenously over 30 min triweekly (Q3W) until disease progression or unmanageable toxicity.

The primary endpoints of this study are objective tumor response, assessed by iRECIST criteria, and time to treatment failure (disease progression). In this regard, patients were clinically and radiologically evaluated at baseline and quarterly after the end of the treatment. Diagnostic imaging included CT, MRI, and Positron Emission Tomography (PET)–CT scan.

To determine the feasibility and toxicity rates of the combined treatment, all patients were clinically and hematologically evaluated, once a week during the treatment course, and quarterly after the end of this. Toxicity data were collected according to CTCAE v4.0.

## 3. Results

From 2020 to 2023, a series of 11 patients with locally advanced or metastatic CSCC were treated at the University of Catania with radiotherapy, either to single or multiple cutaneous lesions, and immunotherapy with cemiplimab. In total, seven patients presented with locally advanced disease, while four patients presented with metastatic disease. Furthermore, four patients presented a single lesion, while five and two patients presented two or more lesions, respectively, up to a total of twenty lesions. Regarding tumor locations, eight (40%) lesions were located in high-risk areas (mask areas of the face, genitalia, hands, and feet), eight (40%) lesions were in medium-risk areas (cheeks, forehead, scalp, neck, and pretibial), and four (20%) lesions were in low-risk areas (trunk and extremities, excluding hands and feet), as reported in Table 1.

All lesions were treated with radiotherapy, accounting for a total of 20 lesions treated, with either CFRT or HFRT.

Regarding radiotherapy, doses and fractionation schedules were tailored based on patients’ conditions and target volumes. In standard conditions, in patients with good performance status and large tumor size (>2 cm), the preferred schedule was CFRT, delivered using a daily dose of 2 Gy, five times a week, up to a total dose of 60 Gy. In the case of high-risk features, dose escalation was performed, up to 66 Gy.

On the contrary, in frail and old patients, those unable to perform daily sessions, or in the case of small lesions (<2 cm), the preferred schedule was HFRT, delivered using a fractional dose of 4–5 Gy, two times a week, up to a total dose of 40–50 Gy (Table 2).

Regarding systemic treatment, all patients received cemiplimab 350 mg, Q3W until disease progression or unmanageable toxicity.

All patients were clinically and hematologically evaluated, once a week during the treatment course and quarterly after the end of this. In terms of early toxicities, only five patients reported grade I–II toxicities, either related to immunotherapy (diarrhea, fatigue, nausea, and constipation) or radiotherapy (radiation dermatitis). No patient reported either early grade III toxicities or late toxicities.

To determine short-term efficacy, patients were clinically and radiologically examined at baseline and quarterly after the end of the treatment, and the response was evaluated according to the iRECIST criteria.

**Figure 1:**
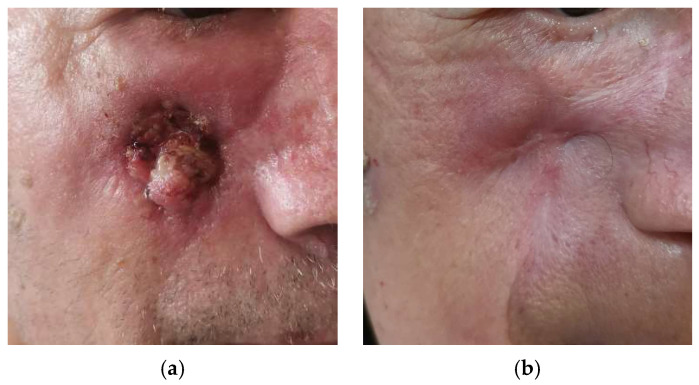
Patient with locally advanced CSCC of the right cheek (area M) at baseline (**a**) and after treatment (**b**).

**Figure 2:**
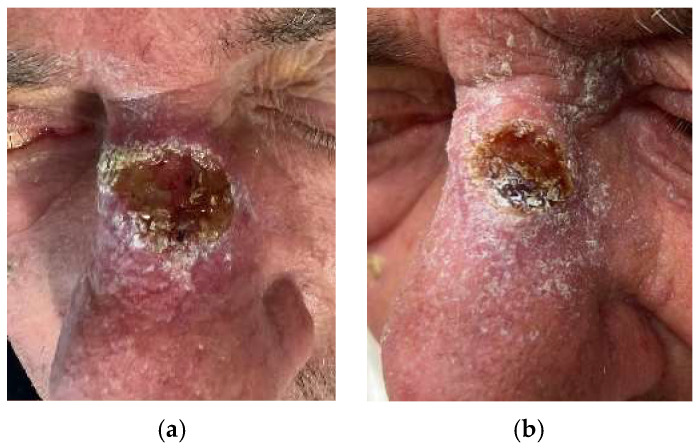
Patient with locally advanced CSCC of the nose (area H) at baseline (**a**) and after treatment (**b**).

**Figure 3:**
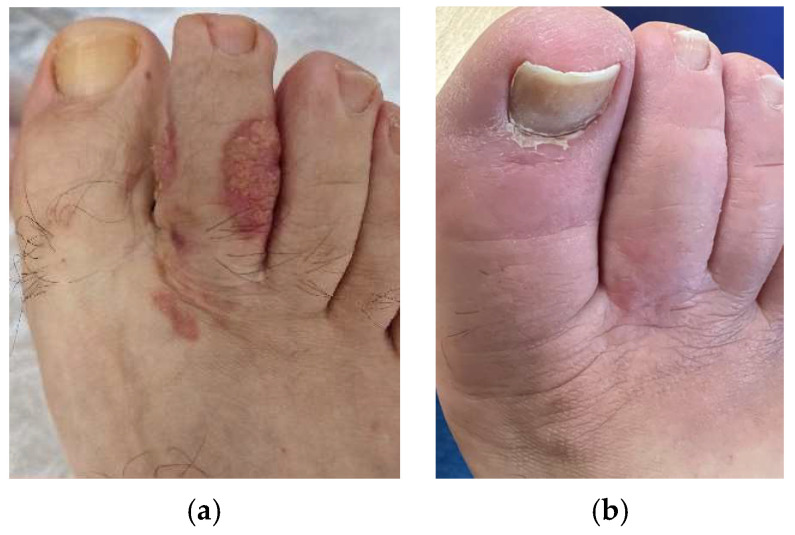
Patient with locally advanced CSCC of the left foot (area H) at baseline (**a**) and after treatment (**b**).

After 6 months, an early clinical and radiological response was observed in 9/11 patients, with 17/20 lesions (85%) presenting either a complete or partial response (Figure 1, Figure 2 and Figure 3). Only 2/11 patients, with a total of 3/20 lesions (15%), had stable disease. The benefits in terms of early response persisted at longer follow-up (21.4 ± 9.7 months), with no patients presenting disease progression.

## 4. Discussion

### 4.1. Theoretical and Practical Bases for Immunotherapy

Even though the incidence of advanced CSCC is increasing, an established consensus on the first-line treatment to administer to this population is still missing [12].

Regarding chemotherapy, even though no agent is currently specifically approved in this setting, cisplatin, either alone or in combination with 5-fluorouracil (5-FU), represents a therapeutic option for patients who require additional treatment beyond surgery and radiotherapy [13]. Regarding EGFR-target therapy, different tyrosine kinase inhibitors (erlotinib, gefitinib) and monoclonal antibodies (cetuximab or panitumumab) have been investigated in the recurrent and metastatic setting [14,15,16,17]. Nevertheless, evidence supporting this approach is still limited.

A major and practice-changing breakthrough in advanced CSCC happened when, in September 2018, the Food and Drug Administration (FDA) approved cemiplimab as an option for patients with metastatic or locally advanced cSCC who are not candidates for surgery or radiotherapy.

In detail, cemiplimab is a high-affinity, highly potent human immunoglobulin G4 (IgG4) anti-PD-1 receptor monoclonal antibody that has demonstrated efficacy in terms of overall response rate and long-term durable response, with a very effective disease control rate in patients with advanced and metastatic CSCC [18]. The mechanism of action is based on the inhibition of PD-1 receptor activity by cemiplimab. Indeed, this recombinant human IgG4 monoclonal antibody binds the receptor, blocking its interactions with programmed cell death ligand-1 and -2 (PD-L1 and PD-L2). The inhibition of the PD-1 pathway results in an immune checkpoint-enhancing T-cell-mediated response, leading to T-cell activation and proliferation against tumor cells [19].

The approval of cemiplimab was based on the results of two early-phase clinical trials that enrolled 163 patients with advanced CSCC in the United States, Australia, and Europe. The first trial was a phase 1 study (NCT02383212) evaluating cemiplimab in patients with advanced malignancies. The second trial was a pivotal phase 2 clinical trial (NCT02760498) specifically assessing cemiplimab in advanced CSCC. Response rates were consistent in both the phase 1 cohorts (50%) and the phase 2 cohort (47%). Additionally, the characteristics of tumor responses demonstrated the efficacy of cemiplimab for treating CSCC in immunocompetent patients. On the other hand, adverse events assessed by investigators were grade 1 or 2 events, with only 8% and 7% of patients in the phase 1 cohorts and the phase 2 cohort discontinuing treatment due to adverse events [18].

Along with cemiplimab, other anti-PD-1 agents are under evaluation in CSCC, such as Pembrolizumab in patients with recurrent/metastatic or locally advanced unresectable CSCC (NCT03284424) and in patients with locally advanced CSCC after surgery and radiation (NCT03833167), and nivolumab as monotherapy in patients with locally advanced/metastatic CSCC (NCT04204837, NCT03834233).

The theoretical bases for the administration of immunotherapy come from the evidence that CSCC has an elevated tumor mutation burden (TMB) and, consequently, is susceptible to higher responsiveness to immune checkpoint inhibitors. Indeed, although skin carcinogenesis is multifactorial, the primary risk factor is represented by prolonged exposure to ultraviolet (UV) radiation, which causes nearly 90% of non-melanoma skin cell cancers [18]. The cumulative exposure to ultraviolet radiation induces different categories of DNA damage in skin cells, such as cyclobutene pyrimidine dimers (CPD), 6-4 photoproducts (6-4 PP), DNA strand breaks, and crosslinks. When these damages cannot be repaired, they become genetic mutations, promoting skin carcinogenesis and increasing TMB load [20,21]. In turn, the accumulation of somatic modification induces the expression of neoantigens (fractions of nonsynonymous mutations that become exposed as epitopes), which can trigger an anticancer response by the immune system. Specifically, neoantigens can activate CD8+ cytotoxic T cells (CTLs) which recognize the peptide bound to major histocompatibility complex class I (MHC I) on tumor cells, and hence initiate tumor cell lysis. Finally, during tumor cell lysis, CTLs release cytotoxic molecules that lead to the destruction of the tumor cell [22].

Given the evidence in the literature that the most robust responses to PD-1/PD-L1 blockade have been seen in tumors with high TMB load, this biomarker and other TMB-derived measures (neoantigens, neoepitopes, and mutation clonality) are all currently under investigation to further stratify patients most likely to respond to immunotherapy.

### 4.2. The Synergistic Effect of Radiotherapy

Even though anti-PD-1 agents have been demonstrated to improve both disease control rate and overall response rate in patients with advanced and metastatic CSCC [18,23], research on how to further enhance SCC outcomes is currently ongoing. In this regard, radiotherapy represents a helpful resource [24,25].

In detail, radiotherapy can provide a synergistic benefit in combination with PD-1 inhibition for patients with CSCC, in terms of both local and distant tumor control, in a T-cell-dependent manner [26,27]. The theoretical basis supporting this combination can be explained through the abscopal effect.

The abscopal effect is a sporadic clinical event that happens when local therapy, typically radiotherapy, induces the shrinkage of a tumoral lesion at a distance from the irradiated site. One of the key components of the abscopal effect is Damage-Associated Molecular Patterns (DAMPs), which play a crucial role in the immune response during tissue damage. In detail, when ionizing radiation induces the death of a tumor cell, the latter induces the release of DAMPs, such as high-motility group box 1 (HMGB1), heat shock proteins (HSPs), calreticulin membrane exposure, and glucose-regulated protein 96 (GP96). In turn, DAMPs promote the activation of dendritic cells through the TLR4 pathway, while HMGB1 promotes dendritic cell maturation, thanks to the pro-inflammatory cytokines (TNF-a, IL-1, IL-6, and IL-8) produced by monocytes [28]. Another important key component is represented by DNA damage induced by ionizing radiation. In detail, DNA fragments that gain access to the cytosol of irradiated cells lead the expression of IFN genes. These genes, in turn, promote the activation of the STING pathway, which enhances anti-tumor CD8+ cells in tumor-draining lymph nodes (TLDNs). Once activated in the TLDNs, tumor-specific CD8 T cells start migrating, guided by inflammatory cytokines and chemokines, extravasating and infiltrating the tumor site. Radiation-induced upregulation of major histocompatibility class I antigens (MHC- I), NKG2D ligands, and death receptors on the cancer cells improves the recognition and killing of the cancer cells by cytotoxic CD8 T cells. In summary, the abscopal effect demonstrates the intricate interplay between local treatments, immune responses, and systemic anti-tumor effects, potentially leading to remarkable clinical outcomes in cancer patients [29].

Even if rare, this effect has been reported in the literature for various types of cancers, including both solid and liquid tumors. Regarding skin cancer, cases of Merkel cell carcinoma and melanoma have been reported in the literature. Regarding CSCC, studies have been investigating the potential immunogenic effect of radiotherapy to stimulate responses in distant nonirradiated sites.

A recently published work demonstrated the benefit of delivering concurrent radiotherapy with cemiplimab, in terms of local effect, but not in terms of distant effect [30]. In detail, this work was the first real-life retrospective study investigating concomitant radiotherapy to cemiplimab as a significant therapeutic strategy. The authors showed that the addition of radiotherapy to cemiplimab enables an earlier clinical–radiological response, with a median duration of 5.5 months in the cemiplimab group versus 3 months in the concomitant therapy group. The response to treatment was prolonged despite discontinuation of cemiplimab, with 91.6% and 83.3% patients in complete or partial remission at 6 months and 1 year after cessation of cemiplimab and no switch to another oncological treatment, respectively. Radiation therapy also provided a therapeutic effect in 83.3% of the patients in the concomitant group, without increasing the occurrence of adverse events [30].

Based on the above-mentioned literature data, we performed a retrospective case series study, investigating the synergistic effect and the immunomodulatory potential of concomitant radiotherapy and immunotherapy. The results of this study (despite its limitations) support the use of this combination to promote objective tumor response and slow disease progression.

Although further investigations are necessary, we can reasonably conclude that the theoretical foundations supporting the synergistic effect of radiotherapy and immunotherapy have the potential to translate into positive clinical outcomes.

## 5. Conclusions

In this retrospective case series, we investigate combination radiotherapy and immunotherapy in 11 patients affected by locally advanced or metastatic CSCC treated at our institution between 2020 and 2023. In total, seven patients presented with locally advanced disease, while four patients presented metastatic disease. Furthermore, four patients presented a single lesion, while three and two patients, respectively, presented two or more lesions, up to a total of twenty lesions. The primary endpoints of this study are objective tumor response, assessed by iRECIST criteria, and time to treatment failure (disease progression).

Despite the retrospective nature of this study, the small sample size, and the lack of a control group, our experience highlights the ability of concomitant radiotherapy and cemiplimab to promote an early clinical–radiological objective response in patients with advanced CSCC, durable with time. Indeed, after 6 months, 85% of CSCC lesions presented either a complete or partial response, while only 15% had stable disease. The benefits in terms of early response persisted at longer follow-up (21.4 ± 9.7 months), with no patients presenting disease progression. No patient reported either early grade III toxicities or late toxicities related to the treatment.

Since local therapy has been delivered to each lesion, it is not possible to conclude whether radiotherapy stimulated the abscopal effect or not. However, we can affirm that it has enhanced early clinical and radiological responses, positively impacting progression-free survival rates.

Even if it is not possible to make practice recommendations, we hope that the results of this study could serve as a basis for designing future larger, randomized, controlled trials, and improve the cooperation between dermatologists, medical oncologists, and radiation oncologists with the common objective of improving patients’ outcomes.

## Figures and Tables

**Table 1:** Patients’ characteristics.

Patients’ Characteristics		%
Sex	Male	63.6%
	Female	36.6%
Age	<65	18.1%
	65–75	36.6%
	>75	45.4%
PS ECOG	0	45.4%
	1	36.6%
	2	9%
	3	0%
Stage	Locally advanced	63.3%
	Metastatic	36.3%
N. of lesions	Single (1)	36.3%
	Multiple (2)	45.5%
	Multiple (≥3)	18.1%
Lesion’s location	High-risk areas	40%
	Medium-risk areas	40%
	Low-risk areas	20%

**Table 2:** Radiotherapy treatment schedules and BED.

Radiotherapy Schedules		BED (α/β = 10)	%
Conventionally fractionated radiotherapy	2–60 Gy	72	15%
	2–66 Gy	79.2	15%
Hypofractionated radiotherapy	4–40 Gy	56	20%
	5–40 Gy	60	40%
	5–50 Gy	75	10%

## Data Availability

The data presented in this study are available on request from the corresponding author. The data are not publicly available due to privacy restrictions.
